# Utility and feasibility of intra-pocket mapping technique for optimal subcutaneous implantable cardioverter defibrillator implantation positioning for R-wave detection: a case series

**DOI:** 10.1093/ehjcr/ytaf348

**Published:** 2025-07-24

**Authors:** Yui Kitami, Satoshi Oka, Kohei Ishibashi, Tomomi Watanabe, Koji Ogawa, Nobuhiko Ueda, Mitsuru Wada, Kengo Kusano

**Affiliations:** Department of Cardiovascular Medicine, National Cerebral and Cardiovascular Center, 6-1 Kishibeshimmachi, Suita, Osaka 564-8565, Japan; Department of Cardiovascular Medicine, National Cerebral and Cardiovascular Center, 6-1 Kishibeshimmachi, Suita, Osaka 564-8565, Japan; Department of Cardiovascular Medicine, National Cerebral and Cardiovascular Center, 6-1 Kishibeshimmachi, Suita, Osaka 564-8565, Japan; Department of Cardiovascular Medicine, National Cerebral and Cardiovascular Center, 6-1 Kishibeshimmachi, Suita, Osaka 564-8565, Japan; Department of Clinical Engineering, National Cerebral and Cardiovascular Center, 6-1 Kishibeshimmachi, Suita, Osaka 564-8565, Japan; Department of Cardiovascular Medicine, National Cerebral and Cardiovascular Center, 6-1 Kishibeshimmachi, Suita, Osaka 564-8565, Japan; Department of Cardiovascular Medicine, National Cerebral and Cardiovascular Center, 6-1 Kishibeshimmachi, Suita, Osaka 564-8565, Japan; Department of Cardiovascular Medicine, National Cerebral and Cardiovascular Center, 6-1 Kishibeshimmachi, Suita, Osaka 564-8565, Japan

**Keywords:** Subcutaneous implantable cardioverter defibrillator, Intra-pocket mapping technique, R-wave amplitude, T-wave over-sensing, Case report

## Abstract

**Background:**

A subcutaneous implantable cardioverter defibrillator (S-ICD) is an alternative to a conventional transvenous implantable cardioverter defibrillator for preventing sudden cardiac death. Although posterior chest S-ICD implantation has been recommended for better defibrillation outcomes, little is known about the optimal S-ICD positioning for R-wave detection. Herein, we report two cases of S-ICD recipients in whom antero-inferior chest positioning improved R-wave detection after posterior chest positioning failed.

**Case summary:**

Two patients experienced intraoperative S-ICD sensing test failures despite passing the preoperative screening. The first case was a 66-year-old man with arrhythmogenic right ventricular cardiomyopathy and superior vena cava syndrome due to transvenous leads. After lead extraction and initial S-ICD placement in the posterior of the mid-chest line, sensing tests failed in all vectors. We performed intra-pocket mapping and repositioning to the antero-inferior chest position, which enabled R-wave detection and a successful defibrillation threshold test. The second case involved a 34-year-old man with short QT syndrome, whose intraoperative sensing tests also failed in all vectors. Antero-inferior repositioning of the S-ICD generator achieved acceptable R-wave detection without T-wave over-sensing and defibrillation threshold test failure.

**Discussion:**

These cases highlight the utility of intra-pocket mapping to optimize S-ICD positioning for R-wave detection in patients with challenging electrocardiogram characteristics. Posterior chest positioning may not be optimal for R-wave detection, particularly in patients with low R-wave and/or high T-wave amplitudes. Repositioning the device closer to the left ventricular apex improved sensing test results, supporting antero-inferior chest placement as a potential solution when posterior chest placement fails.

Learning points

- Generator positioning on or posterior to the mid-chest line is considered optimal to obtain a low PRAETORIAN score and successful defibrillation threshold test.
- We demonstrated cases with intraoperative sensing test failures across all vectors of posteriorly positioned S-ICDs, highlighting the utility and feasibility of the novel intra-generator pocket mapping and relocation of the generator to the slightly antero-inferior position as a bailout technique.
- As compared with defibrillation threshold test success, antero-inferior positioning of the S-ICD generator may be better in terms of sensing test success (R-wave amplitude detection and T-wave over-sensing prevention) in the primary or secondary vectors because of its proximity to the cardiac anatomical axis, a direction from the summit of the inferior pyramidal space towards the left ventricular apex.

## Introduction

Transvenous implantable cardioverter defibrillator (TV-ICD) implantation is an established treatment for preventing sudden cardiac death due to life-threatening ventricular tachyarrhythmias.^1^ An alternative cardiac shock device without pacing function is the subcutaneous implantable cardioverter defibrillator (S-ICD),^2,3^ for which the shock efficacy depends on the device implantation position.^4,5^ This technology, which does not require vascular access, has fewer lead-related complications than conventional TV-ICD.^6^

The PRAETORIAN score—a non-invasive scoring method developed based on clinical and computer modelling—was proposed as a tool for predicting defibrillation threshold (DFT) test success.^7^ Anterior chest positioning has been reported to negatively impact DFT in patients with high PRAETORIAN scores,^8^ making posterior chest positioning the recommended S-ICD implantation position.^9^

We hypothesized that slightly anterior chest positioning may improve R-wave detection adequacy. Herein, we report two cases where S-ICD sensing tests failed in all vectors with posterior positioning despite passing preoperative screening yet succeeded with anterior repositioning via intra-pocket mapping.

## Summary figure

ARVC, arrhythmogenic right ventricular cardiomyopathy; S-ICD, subcutaneous implantable cardioverter defibrillator; SQTS, short QT syndrome; TV-ICD, transvenous implantable cardioverter defibrillator; VT, ventricular tachycardia.

**Figure:**
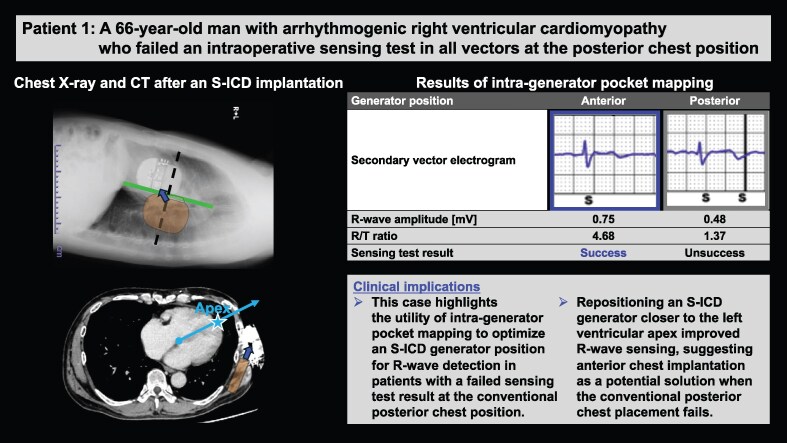


## Patient 1

**Figure 1:**
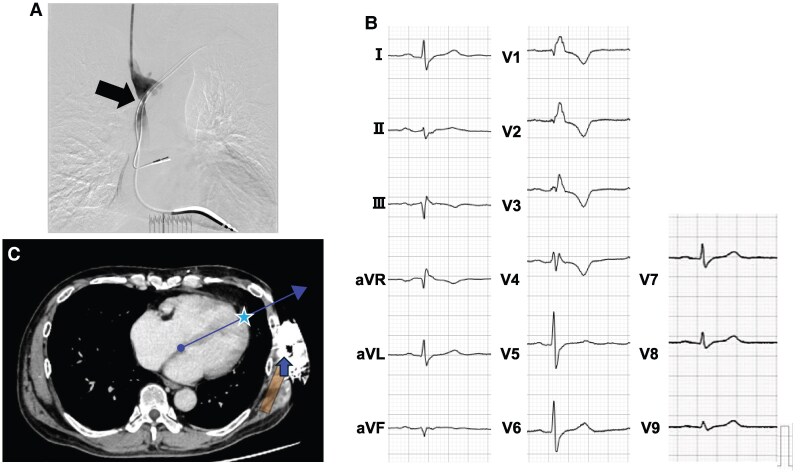
Examinations of Patient 1. (*A*) Venogram showing stenosis of the superior vena cava (arrow) where the transvenous leads were passed. (*B*) Fifteen-lead electrocardiogram showing complete right bundle branch block, low R-wave amplitude, and negative T-wave inversions in leads V1–4. (*C*) Computed tomography image after a subcutaneous implantable cardioverter defibrillator implantation. The cardiac anatomical axis is determined as the line connecting the summit of the inferior pyramidal space (circle) and the apex of the left ventricular apex (star). Subcutaneous implantable cardioverter defibrillator generator relocation to the slightly antero-inferior position improved the detected R-wave amplitude owing to the secondary vector proximity to the cardiac anatomical axis.

A 66-year-old man with arrhythmogenic right ventricular cardiomyopathy (ARVC) had previously received a dual-chamber TV-ICD after catheter ablation for ventricular tachycardia (VT). Two years later, he presented with facial and neck oedema, despite the absence in the legs. Venography revealed superior vena cava (SVC) obstruction where the transvenous leads were passed, leading to a diagnosis of SVC syndrome. There were no remarkable changes in other physical and clinical examinations. Despite repeated plain old balloon angioplasty and anticoagulation therapy, SVC stenosis persisted (*Figure 1A*), with no significant improvement in oedema. Lead extraction and S-ICD implantation were thus indicated. Although the patient’s electrocardiogram showed low R-wave amplitude due to extensive right ventricular myocardial degeneration (*Figure 1B*), the screening test confirmed eligibility for S-ICD implantation within the primary and secondary vectors in both supine and upright positions at a conventional left parasternal lead and posterior chest generator placement.

**Figure 2:**
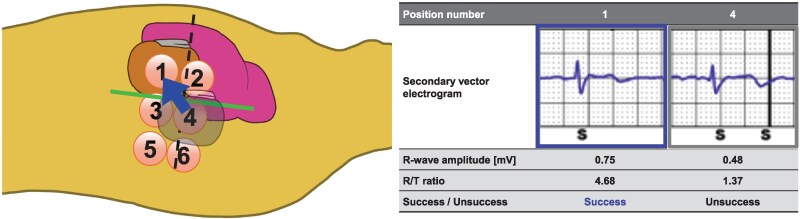
Schematic diagram of intra-pocket electrical mapping and secondary vector electrograms of Patient 1. First, we implanted a generator at Position 4. However, a sensing test failed because of low R-wave amplitude and R/T ratio. We tried intra-pocket electrical mapping at six generator implantation positions. Finally, the highest R-wave amplitude and an R/T ratio were obtained at Position 1. Manually measured R-wave amplitude and R/T ratio at Position 1 were higher than those at Position 4.

**Figure 3:**
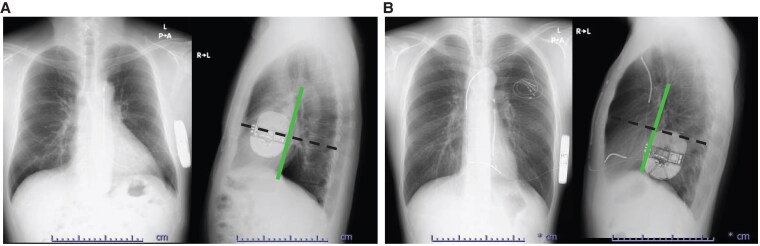
Chest X-rays of postero-anterior and lateral views after subcutaneous implantable cardioverter defibrillator implantation. The subcutaneous implantable cardioverter defibrillator generators are implanted anterior (*A*: Patient 1) and posterior (*B*: Patient 2) from the mid-lateral line (vertical line), respectively. According to the generator positions, the PRAETORIAN scores are different between Patients 1 (120) and 2 (60).

Under general anaesthesia, lead extraction was performed successfully using a locking stylet and a 14 Fr excimer laser sheath. An S-ICD (EMBLEM™, Boston Scientific, Marlborough, MA, USA) was implanted using a two-incision technique, placing the subcutaneous lead and generator in the normal left parasternal and posterior chest positions, respectively (*Figure 2*, Position 4). However, subsequent sensing tests failed across all vectors due to R-wave under-sensing and/or T-wave over-sensing (TWOS), despite a successful preoperative screening test. Thus, repetitive sensing tests were attempted at multiple generator positions in the intra-generator pocket. Finally, the antero-inferior generator replacement just below the incision line (*Figure 2*, Position 1) passed the sensing test in the secondary vector. We then securely fixed the generator to the chest wall to prevent displacement and conducted a DFT test. Despite a PRAETORIAN score of 120 (90–150; intermediate risk of conversion failure; *Figure 3A*), induced ventricular fibrillation was successfully defibrillated with a single 65 J shock after 15 s of detection and charging through the secondary vector. During postoperative treadmill testing, the patient’s peak heart rate was 122 b.p.m., with no double counting in the secondary vector. Chest computed tomography showed the final S-ICD generator implantation position close to the cardiac anatomical axis (*Figure 1C*, blue arrow), determined as the direction from the summit of the inferior pyramidal space, where the atrioventricular node is supposed to be located, towards the left ventricular (LV) apex. Fifteen months post-implantation, the patient remained free of complications or shocks (see Supplementary material online, *Figure S1*).

## Patient 2

**Figure 4:**
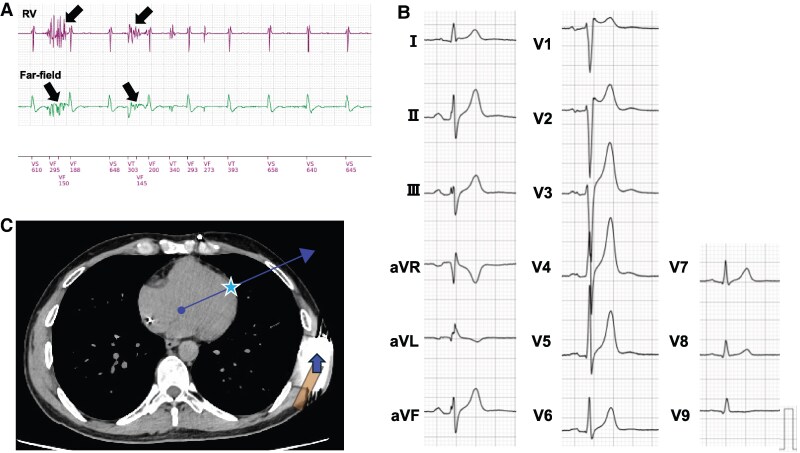
Examinations of Patient 2. (*A*) Intracardiac electrogram of the transvenous implantable cardioverter defibrillator records right ventricular lead noises (arrows) when the patient shook his arms. (*B*) Fifteen-lead electrocardiogram showing a short QTc interval of 330 ms and a high T-wave amplitude. (*C*) Computed tomography image after subcutaneous implantable cardioverter defibrillator implantation. The cardiac anatomical axis is determined as the line connecting the summit of the inferior pyramidal space (circle) and the apex of the left ventricular apex (star). Subcutaneous implantable cardioverter defibrillator generator relocation to the slightly antero-inferior position improved a detected R-wave amplitude owing to the primary vector proximity to the cardiac anatomical axis. RV, right ventricular.

**Figure 5:**
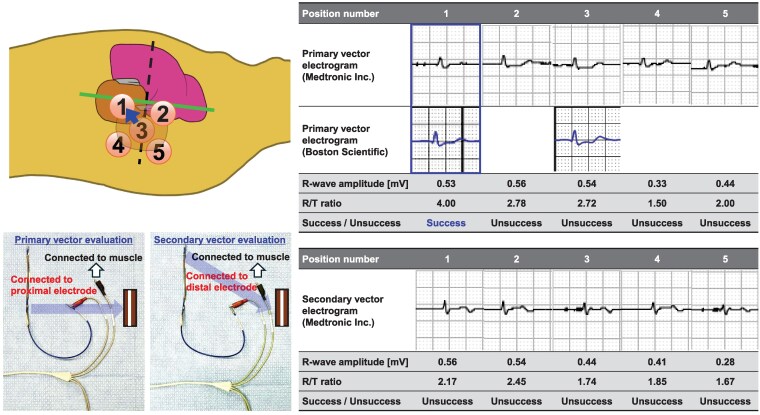
Schematic diagram of intra-pocket electrical mapping and primary and secondary vector electrograms of Patient 2. First, we implanted a generator at Position number 3. However, the sensing test failed due to T-wave over-sensing. We tried intra-generator pocket mapping at five positions using an analyser cable and a pacemaker programmer (Medtronic Inc.). For the evaluation, red-coloured positive clip of the analyser cable was connected to proximal (primary vector) and distal (secondary vector) parts. The black-coloured negative clip of the analyser cable was connected to the serratus anterior muscle of each position numbered from 1 to 5, and the R-wave amplitude and R/T ratio were measured. Finally, an acceptable R/T ratio was obtained, and automatic setup using an S-ICD programmer (Boston Scientific) was passed only at Position number 1 (primary vector).

A 34-year-old man with a family history of sudden death, a history of syncope, and polymorphic VT induced during an electrophysiological study was diagnosed with short QT syndrome (SQTS) and received a TV-ICD for primary prevention. Although he had never experienced appropriate or inappropriate shocks, home monitoring recorded noises from a dual-coil shock lead, suggesting a broken lead (*Figure 4A*). At the time of admission, there were no obvious abnormalities in the physical examination. His electrocardiogram showed a corrected QT interval of 330 ms and high T-wave amplitude (*Figure 4B*). The patient met the criteria for S-ICD implantation and passed its eligibility screening test within the primary vector in both supine and upright positions at a conventional implantation site. Due to the high risk of extracting the dual-coil shock lead, which had been in place for 16 years, we proceeded with TV-ICD generator extraction and S-ICD implantation using a two-incision technique in a standard fashion. The first intraoperative sensing tests failed across all vectors due to TWOS (*Figure 5*, Position 3). Multiple site-sensing tests were conducted in the generator pocket using an analyser cable and pacemaker programmer (Care Link Smart Sync™, Medtronic Inc., Minneapolis, MN, USA). The red-coloured electrode of the analyser cable was connected to the proximal electrode for primary vector evaluation and the distal electrode of the subcutaneous lead for secondary vector evaluation. The R-wave amplitude and R/T ratio were manually measured at five positions in the generator pocket using the black-coloured electrode of the analyser cable, which was directly connected to the serratus anterior muscle (*Figure 5*). A higher R-wave amplitude was obtained in the anterior than in the posterior chest position, and an acceptable R/T ratio was obtained in the lowest site in the anterior chest position. Finally, the sensing test (primary vector) was passed by repositioning the generator to a slightly antero-inferior chest position (*Figure 5*, Position 1) partially within the intermuscular space between latissimus dorsi and serratus anterior muscles. The patient maintained a low PRAETORIAN score (60, <90; low risk of conversion failure; *Figure 3B*) and underwent a successful DFT test, with a single 65 J shock delivered after 19 s of detection and charging through the primary vector. The postoperative treadmill exercise test was passed without double counting due to TWOS. No complications or shocks occurred during the following 14 months (see Supplementary material online, *Figure S1*).

## Discussion

We demonstrated cases of ARVC and SQTS with intraoperative sensing test failures across all vectors of normally positioned S-ICDs, highlighting the utility and feasibility of the novel intra-generator pocket mapping as a bailout technique.

Several factors, such as body mass index and shock impedance, have been reported as predictors of defibrillation success.^5^ However, their odds ratios remain relatively low. The PRAETORIAN score for DFT test success prediction identified the following critical implant position determinants: (i) adipose tissue between the coil and the sternum; (ii) generator malposition along the anterior-posterior axis; and (iii) adipose tissue between the generator and the thorax.^4,10^ The score categorizes the risk of DFT failure into low (<90 points), intermediate (90–150 points), and high (≥150 points).^7^ Recently, a multi-centre study showed that patients with a PRAETORIAN score < 90 have a 99% rate of DFT test success, whereas those with a PRAETORIAN score ≥ 90 have a 25% risk of DFT test failure.^8^ Thus, positioning the generator on or posterior to the mid-chest line is suggested as optimal to obtain a low PRAETORIAN score and DFT test success.^9^

R-wave detection adequacy may also depend on the positioning of the S-ICD system. Data from the multicentre RHYTHM DETECT registry for S-ICD replacement showed that patients who underwent device relocation to the posterior position had a significantly higher prevalence of sensing vector reprogramming using the automatic setup procedure compared with other patients (33% vs. 6%, *P* < 0.001).^11^ Most S-ICD sensing failures are due to R-wave under-sensing and/or TWOS; thus, treatment strategies such as device relocation to increase the detected R-wave amplitude or switching to TV-ICD are necessary. For each of our present patients, although implantation of the system was attempted in the posterior chest position, the sensing test failed for all the vectors.

Subcutaneous implantable cardioverter defibrillator implantation for patients with low R-wave amplitude or high T-wave amplitude is challenging because of the high risk of sensing test failure. The first patient had ARVC, which is known to significantly increase the risk of sensing test failure compared with other cardiomyopathies due to a low R-wave amplitude.^12,13^ The second patient had SQTS, a potassium or calcium channel disorder characterized by a short QT interval and high T-wave amplitude.^14^ Therefore, these cases presented challenges in R-wave detection and/or distinguishing R-waves from T-waves on their electrograms. Despite the limited space initially created, we performed intra-S-ICD generator pocket mapping and relocated the devices to a slightly antero-inferior position. Consequently, both the intraoperative sensing and DFT tests were successful on the primary or secondary vectors.

We speculate that relocating the S-ICD generator to the slightly antero-inferior position improved the detected R-wave amplitude and R/T ratio in the primary or secondary vectors because of its proximity to the cardiac anatomical axis, a direction from the summit of the inferior pyramidal space towards the LV apex. The primary and secondary vectors were determined by the directions from the superior and inferior electrodes towards the S-ICD generator position, respectively. As electrical excitation of the sinus rhythm begins in the sinus node and proceeds towards the apex via the atrioventricular node, the cardiac anatomical axis is correlated with the electrical axis.^15^ Therefore, the maximum R-wave amplitude could be detected at the antero-inferior S-ICD generator position close to the cardiac anatomical axis. Subcutaneous implantable cardioverter defibrillator sensing failure criteria were R-wave amplitude < 0.5 mV and/or R/T ratio < 3.5. In practice, the R-wave amplitude and R/T ratio improved in both cases with antero-inferior replacement (*Figures 2* and *5*). Furthermore, a recent study demonstrated the utility of relocating implantable loop recorders to the apex from the standard left parasternal position to maximize detected R-wave amplitude,^16^ supporting our hypothesis. Despite causing aesthetic and comfort issues, in addition to the risk of DFT failure, the antero-inferior S-ICD generator relocation may be beneficial if the intraoperative sensing tests fail.

This electrophysiological study has several limitations. First, the number of patients whose intraoperative sensing tests failed and subsequently required intra-pocket electrical mapping was limited. At our hospital, all recipients undergo an S-ICD screening test in various positions, and indications are considered before implantation. Therefore, only two cases required the intra-pocket electrical mapping technique. Second, there were limitations to the intraoperative evaluation. Patient 1 was the first case of intraoperative sensing test failure despite preoperative screening success that we encountered. Consequently, electrograms were only recorded at the first and final positions, even though automatic setup evaluations were performed at six generator positions. In Patient 2, the R-wave amplitude was measured using a device programmer from another company, as the number of simultaneously available programmers was limited. However, the automatic set-up results matched the R-wave amplitude evaluation. Third, S-ICD recipients with only one accepted vector were at a risk of inappropriate shock during the follow-up period. Long-term safety remains to be demonstrated, although accurate R-wave detection during the treadmill exercising test was confirmed, and the presented cases have passed over 1 year without an inappropriate shock. Therefore, we considered the intra-generator pocket mapping as a bailout technique. Further investigations are needed to evaluate the association between device-detected R-wave amplitude and S-ICD generator positions and long-term safety after the implantation. In challenging cases, preoperative screening tests might be performed at multiple positions of the axillary electrode, and S-ICD indication should be carefully examined. Finally, subcutaneous lead repositioning might be an alternative bailout technique, as its utility and feasibility cannot be denied. However, re-tunnelling is an invasive procedure; thus, we performed intra-generator pocket mapping to the extent of the limited space initially created, thereby obtaining an optimal R-wave amplitude and R/T ratio non-invasively.

## Conclusions

This study demonstrated the utility and feasibility of the novel intra-generator pocket mapping as a bailout technique in cases of intraoperative S-ICD sensing test failure. Antero-inferior generator relocation may be beneficial when the screening and intraoperative sensing tests fail.

## Supplementary Material

ytaf348_Supplementary_Data

## Data Availability

The data underlying this article are available in the article.

## Lead author biography

Dr Yui Kitami graduated from Kitasato University School of Medicine, Kanagawa, Japan, and subsequently completed her residency in internal medicine at the National Center for Global Health and Medicine, Tokyo. She is now a cardiology fellow at the National Cerebral and Cardiovascular Center, Osaka, with a particular interest in electrophysiology.
