# Supplementary material for: Utility and feasibility of intra-pocket mapping technique for optimal subcutaneous implantable cardioverter defibrillator implantation positioning for R-wave detection: a case series
Source: Eur Heart J Case Rep. 2025 Jul 24;9(8):ytaf348. doi: 10.1093/ehjcr/ytaf348 (PMC12342164; doi:10.1093/ehjcr/ytaf348)
Supplement: ytaf348_Supplementary_Data [file ytaf348_supplementary_data.docx]

**Supplementary figure**

**Timelines of the treatment course of both S-ICD recipients.**

Abbreviations: ARVC, Arrhythmogenic Right Ventricular Cardiomyopathy; S-ICD, Subcutaneous Implantable Cardioverter Defibrillator; SQTS, Short QT syndrome; TV-ICD, transvenous implantable cardioverter defibrillator; VT, Ventricular Tachycardia
